# An Integer Programming Formulation of the Minimum Common String Partition Problem

**DOI:** 10.1371/journal.pone.0130266

**Published:** 2015-07-02

**Authors:** S. M. Ferdous, M. Sohel Rahman

**Affiliations:** 1 Department of Computer Science and Engineering, Ahsanullah University of Science and Technology (AUST), Dhaka, Bangladesh; 2 AℓEDA Group, Department of Computer Science and Engineering, Bangladesh University of Engineering and Technology (BUET), Dhaka, Bangladesh; Technische Universität Dresden, Medical Faculty, GERMANY

## Abstract

We consider the problem of finding a minimum common string partition (MCSP) of two strings, which is an NP-hard problem. The MCSP problem is closely related to genome comparison and rearrangement, an important field in Computational Biology. In this paper, we map the MCSP problem into a graph applying a prior technique and using this graph, we develop an Integer Linear Programming (ILP) formulation for the problem. We implement the ILP formulation and compare the results with the state-of-the-art algorithms from the literature. The experimental results are found to be promising.

## 1 Introduction

In the minimum common string partition (MCSP) problem, we are given two *related* strings (*S*, *T*). Two strings are said to be related if the frequencies of each letter in the two strings match. A partition of a string *S* is defined as a sequence *P* = (*b*_1_, *b*_2_, …, *b*_*c*_), where *b*_*i*_ are substrings of *S* whose concatenation is equal to *S*, i.e., *b*_1_ *b*_2_…*b*_*c*_ = *S*. Given a partition *P* of a string *S* and a partition *Q* of a string *T*, we say that the pair *π* = < *P*, *Q* > is a common partition of (*S*, *T*) if *Q* is a permutation of *P*. The minimum common string partition problem is to find a common partition of (*S*, *T*) with the minimum number of substrings, that is to minimize *c*. For example, if (*S*, *T*) = (**atatgat**,**atgatat**), then an optimal solution is *π* = {**atgat**,**at**} and the minimum common partition size is 2. The restricted version of MCSP where each letter occurs at most *d* times in each input string, is denoted by *d*-MCSP. A more detailed study of the application of MCSP can be found in [1], [2] and [3].

In this paper, we present an Integer Linear Programming (ILP) formulation for the MCSP problem. In particular, we use a graph mapping that was presented in our prior work [4] to solve the MCSP problem using the Ant Colony Optimization technique [5]. Here we exploit this graph to devise an ILP formulation for the problem. Then we implement the ILP formulation, conduct extensive experiments and compare the results with the state-of-the-art algorithms from the literature. As will be reported in a later section, the results clearly indicate that the ILP formulation is effective and provides excellent results. One of the intriguing findings of our work is the fact that our ILP formulation turns out to be more effective and accurate than our meta-heuristics approach presented in [4]. This is especially interesting because both the algorithms are based on the same graph that is constructed through an interesting mapping [4].

The rest of the paper is organized as follows. In Section 2 we present a brief literature review. Section 3 presents the notations and definitions used in this paper. In Section 4 we present the ILP formulation for the MCSP problem. We present our experimental results in Sections 5 followed by a brief relevant discussion in Section 6. Finally, we briefly conclude in Section 7.

## 2 Related Works

The 1-MCSP problem is essentially the breakpoint distance problem [6] between two permutations, which is solvable in polynomial time [1]. The 2-MCSP problem has been shown to be NP-hard and moreover APX-hard in [1]. The authors in [1] also have presented several approximation algorithms to solve the problem. In [2], Chen et al. have studied a generalization of the MCSP problem called the Signed Reversal Distance with Duplicates (SRDD). Furthermore, they have presented a 1.5-approximation algorithm for the 2-MCSP problem. In [7], Damaschke has analyzed the fixed-parameter tractability of the MCSP problem considering different parameters. The MCSP problem is also studied in [8], where it is termed as the true evolutionary distance problem between two genomes. In [9], the authors have investigated the *d*-MCSP problem along with two other variants, namely, *MCSP* ^*c*^, where the alphabet size is at most *c* and *x*-balanced MCSP, which requires that the length of blocks be at most *x* away from the average length. They have shown that *MCSP* ^*c*^ is NP-hard when *c* ≥ 2. As for *d*-MCSP, they have presented an fixed parameter tractable (FPT) algorithm which runs in O*((*d*!)^*k*^) time, where *k* is the number of blocks in the optimal common partition. The result has been improved by Bulteau et al. [10] by showing that MCSP can be solved in *O*(*d* ^2*k*^ ⋅ *kn*) time. Recently, Bulteau and Komusiewicz [11] have introduced the first fixed-parameter algorithm for the MCSP problem using parameter *k* only.

Chrobak et al. [3] have analyzed a natural greedy heuristic for the MCSP problem: iteratively, at each step, it extracts a longest common substring from the input strings. They have shown that for the 2-MCSP problem, the approximation ratio (for the greedy heuristic) is exactly 3. They also have proved that for the 4-MCSP problem the ratio is log *n* and for the general case, it lies between Ω(*n* ^0.43^) and *O*(*n* ^0.67^). In [12], He has proposed an improved greedy algorithm based on the greedy strategy of [3], where the idea is to extract the longest common substring containing a symbol occurring only once at each step whenever there is such a symbol.

In our prior work [4], we have developed a meta-heuristc algorithm, namely, MAX-MIN ant system to solve the MCSP problem. In particular, in [4], we have mapped the instance of the MCSP problem into a graph, namely, the common substring graph. MAX-MIN Ant System has been implemented over this graph. Recently in [13], Blum et al. have proposed an iterative probabilistic tree search algorithm for solving this problem. The algorithm is an iterative probabilistic variant of the greedy algorithm of [3]. The authors have tested their approach with the dataset introduced in [4]. Subsequently, a common block based ILP formulation has been proposed in [14] by Blum et al. They have tested their ILP formulation on the previous benchmarks [4] as well as on a new benchmark of 7 larger instances.

## 3 Preliminaries

This section summarizes the definitions and notations used throughout the paper. Two strings (*S*, *T*), of equal length (*n*), over an alphabet ∑ are called *related* if the frequencies of the letters in the two strings match. We define a block *B* = [*S*, *i*, *j*], 0 ≤ *i* ≤ *j* < *n*, of a string *S* as a data structure where *i* and *j* denote the starting and ending positions of the block. A block, [*S*, *i*, *j*] represents a substring of *S* denoted as *substring*([*S*, *i*, *j*]) with length (*j* − *i*+1).

As an example, if we have two strings (*S*, *T*) = (**atgcat**,**tgcata**), then [*S*, 0, 1] and [*S*, 4, 5] both represent the substring **at** of *S*. In other words, *substring*([*S*, 0, 1]) = *substring*([*S*, 4, 5]) = **at**. We say that a block *B* matches with another block *B*′ if the two blocks represent the same substrings. Given a list of blocks *l*_*b*_, *matchList*(*l*_*b*_, *B*) is defined as a list of those blocks of *l*_*b*_ that match *B*. For the example stated above, let a list of blocks be *l*_*b*_ = {[*S*, 0, 1], [*S*, 1, 1], [*S*, 4, 5]} and *B* = [*S*, 0, 1]; then *matchList*(*l*_*b*_, *B*) = {[*S*, 0, 1], [*S*, 4, 5]}.

We use the notion of a common substring graph as introduced in [4]. A common substring graph, *G*_*cs*_(*V*, *E*, *S*) of two strings (*S*, *T*) is defined as follows. Here *V* is the vertex set of the graph and *E* is the edge set. Vertices are the positions of string *S*, i.e., for each *v* ∈ *V*, *v* ∈ {0, *n* − 1}. Two vertices *v*_*i*_ ≤ *v*_*j*_ are connected with an edge, i.e, (*v*_*i*_, *v*_*j*_) ∈ *E*, if the substring induced by the block [*S*, *v*_*i*_, *v*_*j*_] matches some substring of *T*. More formally, if *S*_*T*_ denotes the set of all substrings of *T*, we have:

$$\left(v_{i},v_{j}\right)\in E\Leftrightarrow \exists s\in S_{T}\;:\;substring\left(\left[S,v_{i},v_{j}\right]\right)=s\;\;\;\;$$

In other words, each edge in the edge set corresponds to a *block* satisfying the above condition. For convenience, we will denote the edges as *edge blocks* and use the list of edge blocks (instead of edges) to define the edge set *E*.

For example, suppose (*S*, *T*) = (**atgcta**,**atgcat**). The corresponding common substring graph of the first string *S*, denoted by *G*_*cs*_(*V*, *E*, *S*), will have vertex set, *V* = {0, 1, 2, 3, 4, 5} and edge set, *E* = {[*S*, 0, 0], [*S*, 1, 1], [*S*, 2, 2], [*S*, 3, 3], [*S*, 4, 4], [*S*, 5, 5], [*S*, 0, 1], [*S*, 1, 2], [*S*, 2, 3], [*S*, 0, 2], [*S*, 1, 3], [*S*, 0, 3]}.

## 4 ILP Formulation

Suppose we are given two related strings (*S*, *T*), each of length *n*. We create two graphs, namely, *G*_*cs*_(*V*_1_, *E*_1_, *S*) and *G*_*cs*_(*V*_2_, *E*_2_, *T*) of (*S*, *T*), where *V*_1_ and *V*_2_ are the vertex sets and *E*_1_ and *E*_2_ are the edge block sets of the two graphs respectively. We define two sets of binary variables, namely, *x*_*t*_1__ and *y*_*t*_2__ where *t*_1_ ∈ *E*_1_ and *t*_2_ ∈ *E*_2_. We also write *δ*_*k*_(*v*)^−^ and *δ*_*k*_(*v*)^+^ for the sets of incoming and outgoing edge blocks from *E*_*k*_ where *v* ∈ *V*_*k*_ and *k* ∈ {1, 2}. An incoming (outgoing) edge block is the one whose starting (ending) position *i* (*j*) is 0 (*n* − 1). With the above setting, we develop an ILP formulation (denoted as ILP_graph_) for the MCSP problem using the common substring graph as follows:

$$\text{minimize}\;\sum_{t_{1}\in E_{1}}x_{t_{1}}$$ (1)

$$\text{subject}\;\text{to}\;\sum_{t_{1}\in E_{1}}x_{t_{1}}=\sum_{t_{2}\in E_{2}}y_{t_{2}}$$ (2)

$$\sum_{t_{1}\in \delta_{1}{\left(0\right)}^{+}}x_{t_{1}}=1$$ (3)

$$\sum_{t_{1}\in \delta_{1}{\left(v\right)}^{-}}x_{t_{1}}=\sum_{t_{1}\in \delta_{1}{\left(v+1\right)}^{+}}x_{t_{1}}\;\;\forall v\in [0,n-1]$$ (4)

$$\sum_{t_{2}\in \delta_{2}{\left(0\right)}^{+}}y_{t_{2}}=1$$ (5)

$$\sum_{t_{2}\in \delta_{2}{\left(v\right)}^{-}}y_{t_{2}}=\sum_{t_{2}\in \delta_{2}{\left(v+1\right)}^{+}}y_{t2}\;\;\forall v\in [0,n-1]$$ (6)

$$\sum_{b_{1}\in matchList\left(E_{1},t_{1}\right)}x_{b_{1}}=\sum_{b_{2}\in matchList\left(E_{2},t_{1}\right)}y_{b_{2}}\;\;\forall t_{1}\in E_{1}$$ (7)

$$x_{t_{1}}\in \left\{0,1\right\},y_{t_{2}}\in \left\{0,1\right\}$$ (8)

### 4.1 Explanation of the Formulation

#### Objective function

Eq 1 is the objective function that is to be minimized. The function simply calculates the size of the partition.

#### Equality constraint

Eq 2 states that two partitions on the two substring graphs must be of equal size. In other words, the number of blocks in the factorization of the first string *S* must be equal to the number of blocks in the factorization of the second string *T*.

#### Factorization constraint

Eqs 3 and 4 together ensures that a unit flow enters at the source (the vertex labelled with 0) and arrives at the sink (the vertex labelled with *n* − 1) for string *S*. So, the string is factorized. For string *T* the factorization is achieved in a similar fashion by Eqs 5 and 6. These constraints ensure that the strings get factorized by non-overlapping blocks.

#### One to one match constraint

We have two sets of blocks after the factorization. We must ensure that there is a one to one matching between the two sets of blocks. By matching we mean that, for each selected block (with *x*_*t*_ = 1 where *t* ∈ *E*_1_) of the first edge block set *E*_1_, there must be one and only one corresponding selected block (with *y*_*t*_ = 1 where *t* ∈ *E*_2_) with the same substring in the second edge block set *E*_2_ and vice versa. Eq 7 achieves the one to one matching by ensuring that for each edge block, the number of selected blocks in *E*_1_ equals the number of selected blocks in *E*_2_.

#### Integrality constraint

Eq 8 ensures the integrality of the variables.

This is a polynomial formulation. The number of variables as well as the number of constraints of the formulation depends on the size of the edge block sets, *E*_1_ and *E*_2_. In the worst case, the number of variables and constraints can be *O*(*n* ^2^), where *n* is the size of the vertex set. But in practice the number of variables is much less than that which is evident from the experimental results as reported in the following section.

## 5 Experiments

Except for one, we have conducted all our experiments in a computer with Intel(R) Core(TM) i5-2450M CPU @2.50 GHz having an installed memory (RAM) of 4.00 GB. There is one particular experiment that has been run in another machine with the same configuration except that the available RAM was higher, 8.00 GB. The operating system was Windows 8.1. The programming environment was Matlab. We have used SCIP (version 3.1.0) standalone solver [15] to solve ILP_graph_.

### 5.1 Data sets

We have conducted our experiments on 5 sets of random synthetic data (henceforth labelled as Group1-Group5) and a real gene sequence dataset (henceforth labelled as Real). The datases are briefly described below.

#### Group1-Group3

In our previous work [4], we generated uniform random DNA sequences, each of length at most 600, using “FaBox (1.41)” [16]. A pair of DNA sequences (*S*, *T*) was generated by randomly shuffling [16] one DNA sequence from the set using “Sequence Manipulation Suite” [17]. This dataset is divided into 3 groups. The first 10 (Group1) have lengths less than or equal to 200 bps (base-pairs), the next 10 (Group2) have lengths within [201, 400] and the rest 10 (Group3) have lengths within [401, 600] bps. Notably, these datases are also used for experimentation and analysis by researchers in recent papers [13, 14].

#### Group4

We have also tested our formulation with a new random dataset collected through personal communication with Christian Blum, one of the co-authors of [14]. This new dataset is a collection of 300 uniform random instances of different lengths and alphabet sizes. The sequences in the dataset are of lengths {100, 200, 300, 400, 500, 600, 700, 800, 900, 1000} and of alphabet size {4, 12, 20}. In particular, for each length there are 30 sequences among which the first 10 are of alphabet size 4, the next 10 are of alphabet size 12 and the rest are of alphabet size 20.

#### Group5

This dataset was introduced in [14] to test the solving limit of their ILP formulation. This constitutes 7 instances of length {800, 1000, 1200, 1400, 1600, 1800, 2000}.

#### Real

We have used the real gene sequence data used in [4]. This data correspond to the first 15 gene sequences of Bacterial Sequencing (part 14) whose lengths are within [200, 600].

### 5.2 Implementation

SCIP [15] (version 3.1.0) standalone solver is used to solve the ILP formulation. SCIP runs on single thread [18]. The solution of an instance is a two steps procedure. Firstly for each instance we have to generate the variables and constraints in a format that is understandable to SCIP. Using Matlab we have generated the MPS (Mathematical Programming System) files of the instances. These files are the input to the solver. For the solver, we have enforced a time limit of 3600 cpu seconds for Group1-Group3, Group4 and Real. The First 5 out of the 7 instances of Group5 have been allowed 3600 seconds each whereas the other 2 have been given 7200 seconds each. All other parameters have been left default.

### 5.3 Results and Analysis

In an updated and extended version [19] (the preprint is available at [20]) of our earlier work [4], MAX-MIN ACO (referred to as MMAS henceforth) has been compared with the greedy algorithm of [3]. In [13], the authors have compared their two versions of iterative probabilistic tree search (TS1 and TS2) with Greedy and MMAS. Here we report only the best of the two tree search solutions (henceforth referred to as TS). Recently in [14], the authors have compared the results of their ILP formulation (ILP_orig_) with Greedy, MMAS and TS. Here, we compare our ILP formulation, i.e., ILP_graph_ with MMAS [4, 19, 20], TS [13] and ILP_orig_[14]. As for the greedy algorithm, we have considered the improved greedy approach in [12] (henceforth labelled as Greedy).

Table 1 presents the comparison among the results of ILP_graph_ and other competitive approaches for Group1-Group3 and Real dataset. For each group the first column is the instance number. The second, third and forth columns represent the common partition size by Greedy [12], MMAS [19] and TS [13] respectively. The fifth to eighth column summarize the results of ILP_orig_. The result is obtained from [14]. The fifth column is the partition size. The sixth column is the time in second, presented as X/Y format only when the solver has been unable to find the optimal solution in 3600 cpu seconds; otherwise it is shown as a single value format reporting the time to get the optimal solution. The seventh column report the relative gap, where gap is defined as the difference between the value of the best valid solution (primal bound) and the lower bound (dual bound) of the problem. The relative gap is formulated as ∣(*upperbound* − *lowerbound*)/*min*(∣*upperbound*∣, ∣*lowerbound*∣)∣. The eighth column is the number of variables in the formulation for the instance. The last four columns report the result of our formulation, ILP_graph_. The columns here reports the same information as the fifth to eighth columns. The best result for an instance is boldfaced.

**Table 1:** Comparison for Group1-Group3 and Real dataset.

					ILP_orig_				ILP_graph_			
	Instance no.	Greedy	MMAS(Avg.)	TS(Avg.)	value	time(sec.)	gap (%)	#vars	value	time(sec.)	gap(%)	#vars
Group1	1	46	42.87	42.30	**41**	1	0	4299	**41**	6.01	0	781
	2	56	51.87	48.90	**47**	3	0	6211	**47**	16.00	0	928
	3	62	57.00	56.00	**52**	30	0	8439	**52**	24.06	0	1172
	4	46	43.33	43.00	**41**	2	0	4299	**41**	12.69	0	736
	5	44	42.93	41.00	**40**	1	0	4718	**40**	5.42	0	833
	6	48	42.80	41.10	**40**	3	0	4435	**40**	14.87	0	765
	7	65	60.60	60.80	**55**	38	0	8687	**55**	81.75	0	1159
	8	51	46.93	45.30	**43**	3	0	4995	**43**	14.42	0	816
	9	46	45.53	43.00	**42**	2	0	4995	**42**	14.31	0	767
	10	63	59.73	58.80	**54**	50	0	9699	**54**	149.84	0	1254
	Avg.	52.7	49.36	48.02	**45.5**	13.3	0	6077.7	**45.5**	33.937	0	921.1
Group2	1	119	113.93	112.1	**98**	66/1969	2.9	37743	99	11.05/511.74	3.78	2740
	2	122	118.93	115.6	106	129/1032	7.5	47174	**104**	14.55/1709.76	5.40	3191
	3	114	112.53	107.6	**97**	55/1216	2.7	36979	**97**	12.35/1382.78	2.63	2776
	4	116	116.40	112.4	102	63/949	4.9	40960	**101**	12.42/487.63	3.42	2914
	5	135	132.20	128.7	116	146/3299	6.7	52697	**115**	16.91/1422.04	5.85	3291
	6	108	106.07	103.2	**93**	56/1419	5.6	35650	94	16.30/3209.79	6.29	2694
	7	108	98.40	96.7	**88**	41/2776	6.0	30839	**88**	8.19/936.17	6.09	2494
	8	123	118.40	115.1	**104**	101/2980	5.1	42668	**104**	11.18/427.08	5.06	2954
	9	124	119.47	114.5	104	81/1630	5.2	42998	**103**	10.56/2962.39	4.19	2924
	10	105	101.87	98.6	89	32/1458	3.6	31169	**88**	8.18/2232.43	1.99	2423
	Avg.	117.4	113.82	110.45	99.7	77/1873	5.02	39887.7	**99.3**	12.17/1528.18	4.47	2840.1
Group3	1	182	179.93	172.9	**155**	733/1398	7.50	110973	**155**	56.98/257.47	8.10	5230
	2	175	176.20	170.7	155	553/869	7.70	102670	**152**	36.98/1202.47	5.56	4849
	3	196	187.87	186.3	166	746/2183	8.50	119287	**161**	55.67/564.16	5.69	5339
	4	192	184.27	180.5	**159**	731/1200	6.90	114975	**159**	332.4/2218.22	7.38	5251
	5	176	171.53	164.7	150	485/886	9.70	99775	**148**	45.35/1089.34	9.17	4917
	6	170	163.47	164.4	147	399/764	9.10	88839	**146**	31.7/468.41	7.99	4441
	7	173	168.47	162.4	149	524/990	9.80	95765	**148**	34.92/2389.80	7.55	4734
	8	185	176.33	171.9	151	492/3584	6.70	97400	**150**	35.48/2654.92	6.10	4691
	9	174	172.80	170.4	158	571/1186	10.90	104186	**154**	40.46/1459.22	9.25	5009
	10	171	167.20	162.3	148	547/1446	9.10	98237	**146**	47.38/1387.57	8.37	4823
	Avg.	179.4	174.81	170.65	153.8	578/1451	8.59	103210.7	**151.9**	71.73/1369.16	7.52	4928.40
Real	1	95.00	87.67	87.30	**78**	972	0.00	22799	**78**	2.26/653.99	0.00	1966
	2	161.00	156.33	154.50	139	432/752	9.20	80523	**136**	37.45/1563.62	7.10	4330
	3	121.00	117.07	113.80	104	125/3580	5.60	45869	**103**	14.61/1888.26	4.58	3052
	4	173.00	164.87	160.30	144	577/1730	6.50	91663	**142**	41.06/1811.39	5.16	4467
	5	172.00	171.07	167.60	150	778/2509	7.90	108866	**149**	83.75/1321.63	7.89	5068
	6	153.00	146.00	144.10	128	257/3578	6.50	70655	**127**	52.03/2108.05	6.07	3836
	7	140.00	141.00	132.50	121	359/2187	6.90	73502	**120**	30.91/589.77	6.23	4187
	8	134.00	133.13	128.70	**116**	275/3365	6.80	65560	117	21.23/1114.30	7.91	3879
	9	149.00	147.53	142.60	131	399/613	8.80	75833	**128**	22.38/676.10	6.56	4130
	10	151.00	150.53	145.30	131	311/1771	7.20	69560	**128**	19.59/1897.79	4.55	3876
	11	126.00	125.00	121.60	**110**	205/3711	4.80	56160	**110**	16.00/3314.21	4.98	3546
	12	143.00	139.13	139.00	126	299/793	9.80	70861	**123**	22.30/474.68	7.83	3981
	13	180.00	181.53	173.20	156	784/1130	7.10	115810	**155**	77.87/883.82	6.58	5251
	14	152.00	149.33	147.30	134	370/2456	9.70	73449	**131**	34.52/429.76	7.84	3905
	15	157.00	161.60	153.10	**139**	560/1762	7.70	91060	**139**	42.15/2137.48	8.18	4556
	Avg.	147.13	144.79	140.73	127.13	409/2131	6.97	74144.67	**125.73**	34.54/1390.99	6.10	4002

**Fig 1:**
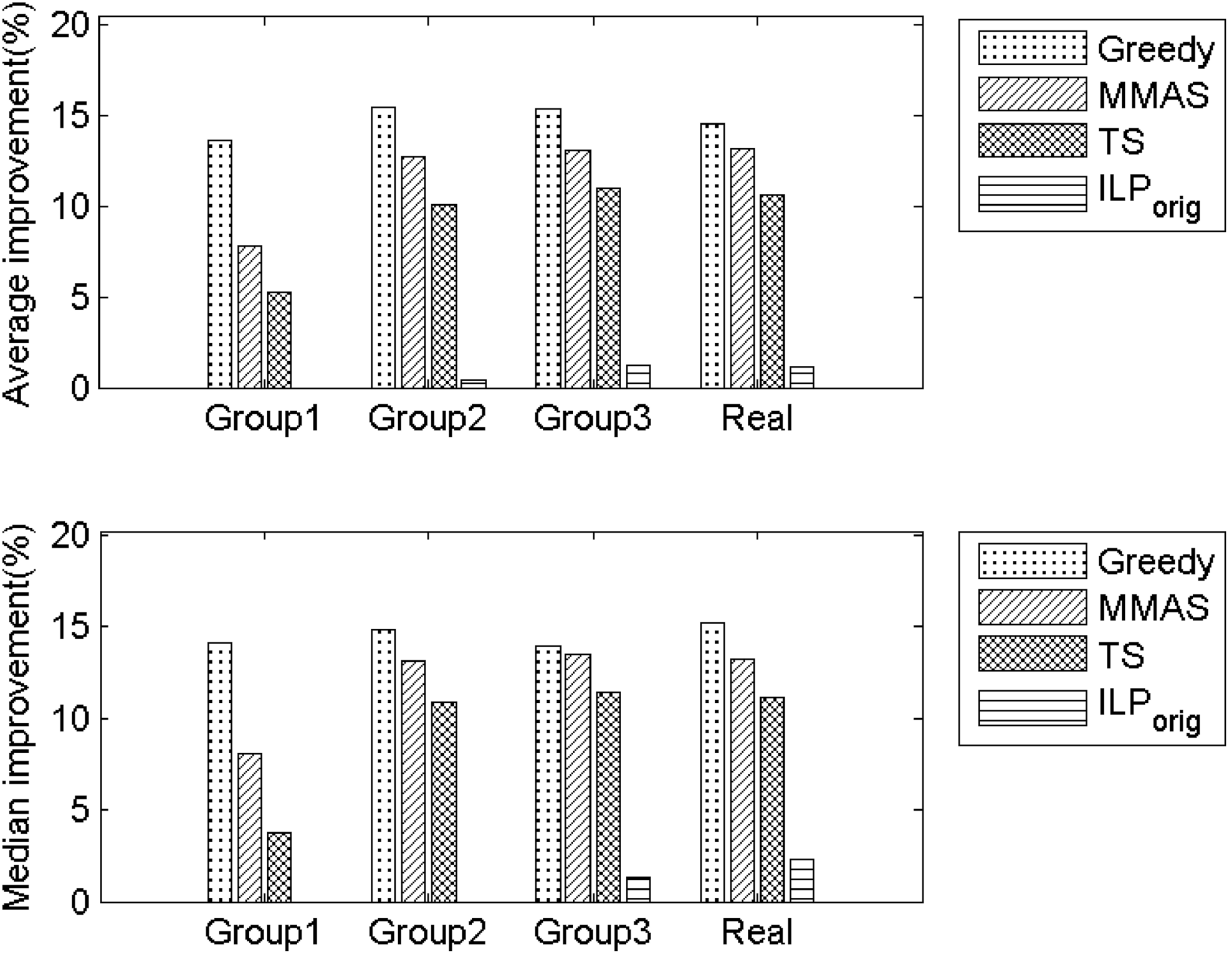
Percentage of improvement of ILP_graph_ over Greedy, MMAS, TS and ILP_orig_. Top: Improvement in average solution. Bottom: Improvement in median solutions.

From Table 1, it is easily verified that ILP_graph_ provides much better common partition size than other approaches. Out of 45 instances, it provides equal or better partition size than ILP_orig_ in 42 cases, amongst which 23 are strictly better. The improvement is not only in the solution size but also in computational time. Except for Group1, ILP_graph_ has been able to achieve improved solution in significantly less time than ILP_orig_. The number of variables are also dramatically reduced in ILP_graph_. Fig 1, shows the percentage of improvement of ILP_graph_ over the other five approaches considered. The significant improvement can be perceived from the figure.

Table 2 reports the average results of Group4 dataset. Here the average of the results of 10 instances for each length group having a particular alphabet size is reported. For example, the first row reports the average results of ten 100-length instances on an alphabet size of 4. The result of ILP_orig_ is collected through personal communication with the author of [14]. It is notable that for the Group4 dataset, ILP_orig_ was implemented using GCC 4.7.3 and IBM ILOG CPLEX V12.1. Moreover, as reported in [14], the corresponding experiments were conducted on a cluster of PCs with 2933MHz Intel(R) Xeon(R) 5670 CPUs having 12 nuclei and 32GB RAM. The third to seventh columns report the solution of ILP_orig_ while the eighth to thirteenth columns report the solution of ILP_graph_. The columns report the same information as in Table 1 with four exceptions as follows. Firstly, the time when the first valid solution is achieved and the time when the best solution is achieved within the time limit (3600 sec) are presented in two different columns (labelled as *ftime* and *time* respectively). Secondly, for each formulation, how many among the 10 instances (represented by each row) have been solved optimally is reported in the column named *#opt*. Finally, the last two columns represent the percentage of improvement in average partition size and the percentage of decrease in the number of variables of ILP_graph_ over ILP_orig_ respectively.

**Table 2:** Comparison of average results on Group4 dataset.

		ILP_orig_						ILP_graph_							
length	alphabet Size	value	ftime	time	#opt	gap	#vars	value	ftime	time	#opt	gap	#vars	%impr(sol)	%impr(var)
100	4	**37.3**	0	0	10	0.00	3425.6	**37.3**	0.17	5.04	10	0.00	649.7	0.00	**81.03**
	12	**68.5**	0	0	10	0.00	993.3	**68.5**	0.04	0.05	10	0.00	324.0	0.00	**67.38**
	20	**79.8**	0	0	10	0.00	622.4	**79.8**	0.02	0.02	10	0.00	264.2	0.00	**57.55**
200	4	**63.5**	3	101	10	0.00	13498.5	**63.5**	2.67	210.75	10	0.00	1473.8	0.00	**89.08**
	12	**119.2**	0	0	10	0.00	3824.6	**119.2**	0.23	0.59	10	0.00	762.8	0.00	**80.06**
	20	**146.2**	0	0	10	0.00	2301.1	**146.2**	0.08	0.08	10	0.00	591.6	0.00	**74.29**
300	4	**88.5**	21	2358	1	3.20	30398.5	88.6	58.76	1455.52	1	3.23	2412.5	-0.11	**92.06**
	12	**165.3**	1	3	10	0.00	8478.6	**165.3**	1.04	17.49	10	0.00	1249.1	0.00	**85.27**
	20	**206.7**	0	0	10	0.00	5029.6	**206.7**	0.19	0.23	10	0.00	967.0	0.00	**80.77**
400	4	115.5	89	2159	0	6.70	53658.5	**114.3**	28.67	1709.47	0	5.65	3369.8	**1.04**	**93.72**
	12	**208.9**	3	47	10	0.00	14887.2	**208.9**	3.61	73.09	10	0.00	1742.1	0.00	**88.30**
	20	**261.5**	1	1	10	0.00	8932.0	**261.5**	0.60	0.73	10	0.00	1366.8	0.00	**84.70**
500	4	139.3	192	870	0	9.10	84004.2	**135.8**	43.85	1306.94	0	6.84	4411.8	**2.51**	**94.75**
	12	**249.0**	10	328	10	0.00	23173.1	**249.0**	6.04	500.94	10	0.00	2266.2	0.00	**90.22**
	20	**312.2**	4	4	10	0.00	13761.0	**312.2**	1.35	4.08	10	0.00	1803.3	0.00	**86.90**
600	4	162.2	487	1893	0	9.40	120795.1	**159.0**	131.19	2018.26	0	7.94	5451.3	**1.97**	**95.49**
	12	291.0	32	1202	2	0.90	33372.6	**290.9**	19.28	1703.27	**4**	0.48	2780.3	**0.03**	**91.67**
	20	**362.3**	6	12	10	0.00	19543.8	**362.3**	3.34	14.61	10	0.00	2253.2	0.00	**88.47**
700	4	187.7	785	2856	0	10.00	164116.2	**182.6**	179.51	1475.00	0	7.93	6459.3	**2.72**	**96.06**
	12	331.0	54	1811	0	1.20	45303.9	**330.9**	11.83	2404.24	0	0.99	3312.0	**0.03**	**92.69**
	20	**408.9**	12	120	10	0.00	26588.5	**408.9**	5.03	70.57	10	0.00	2729.3	0.00	**89.74**
800	4	221.6	1442	3432	0	14.70	213956.1	**207.7**	349.98	1674.61	0	10.40	7555.9	**6.27**	**96.47**
	12	**368.7**	123	2460	0	1.60	59026.8	369.5	17.87	1936.50	0	1.71	3871.0	-0.22	**93.44**
	20	**456.1**	33	669	10	0.00	34451.6	**456.1**	12.09	489.33	10	0.00	3180.1	0.00	**90.77**
900	4	266.3	1880	2314	0	22.30	271158.3	**227.7**	491.91	2315.87	0	10.12	8682.5	**14.49**	**96.80**
	12	408.5	178	2406	0	2.20	74372.5	**407.9**	26.34	1936.12	0	1.96	4440.8	**0.15**	**94.03**
	20	**501.5**	50	1625	6	0.20	43543.4	**501.5**	11.44	1311.72	**7**	0.15	3649.8	0.00	**91.62**
1000	4	288.7	3253	3739	0	21.80	334125.1	**249.2**	540.01	1752.42	0	10.49	9825.4	**13.68**	**97.06**
	12	449.2	306	3147	0	2.90	91955.2	**445.4**	21.53	1784.16	0	1.89	5017.2	**0.85**	**94.54**
	20	546.9	89	2182	1	0.50	53736.0	**546.7**	9.99	1224.10	**5**	0.29	4106.7	**0.04**	**92.36**

Like Group1-Group3 and Real dataset, the results of Table 2 draw the same conclusion. The ILP_graph_ formulation provides better solutions than ILP_orig_ in almost every aspect. Numerically, ILP_graph_ gets equal or better average partition in 28 out of 30 instances of which 12 are strictly better. The number of instances solved optimally by ILP_graph_ is 172 (out of 300) which is 12 more than that of ILP_orig_. The percentage of improvements in the average solutions also proves the superiority of ILP_graph_. As it is evident from Table 2, the improvement gets more acute with the increase of the string length and the decrease of the alphabet size. This observation is also supported by Table 3 that reports the solutions of the two formulations for Group5 dataset. The 7 instances of Group5 were introduced in [14] to test the limit of their formulation. Their simulation [14] was conducted in a cluster of PCs with “Intel(R) Xeon(R) CPU 513” CPUs of 4 nuclei of 2000 MHz and 4 Gigabyte of RAM with the time limit of 12 hours. On the other hand, for this dataset, we have enforced 3600 seconds for the first 5 instances and 7200 seconds for the last two. ILP_orig_ could not achieve a valid solution for the last instance even within 12 hours whereas ILP_graph_ got a valid solution in 6100 seconds. From the percentage of improvement (*%impr*) it can be concluded that, ILP_graph_ achieves better partition size with less time as the length of the string increases. The number of variables also become intractable for ILP_orig_ as the length increases. All of these results speak in favor of ILP_graph_.

**Table 3:** Comparison result for Group5 dataset. NSF means “No solutions found”.

	ILP_orig_				ILP_graph_					
length	value	time	#vars	gap	value	time	#vars	gap	%impr(sol)	%impr(var)
800	210	3228	214622	10.70	**204**	2701	7546	8.70	**2.86**	**96.48**
1000	304	2922	334411	26.40	**245**	2964	9909	9.43	**19.41**	**97.04**
1200	342	6220	480908	22.60	**306**	850	11947	15.58	**10.53**	**97.52**
1400	401	12124	653401	24.90	**343**	3433	14316	13.95	**14.46**	**97.81**
1600	442	20616	854500	24.10	**381**	3600	16677	13.65	**13.80**	**98.05**
1800	486	37304	1084533	24.00	**420**	4723	19275	13.65	**13.58**	**98.22**
2000	NSF	NSF	1335893	NSF	**314**	6100	21494	13.95	NA	NA

Finally, to further test the limit of our formulation, i.e., ILP_graph_, we have conducted an experiment with an instance of length 3000 on the machine with 8GB of RAM. The time limit was set to 12 hours. ILP_graph_ has been able to get a valid solution of partition size 642 in 11 hours.

### 5.4 Running Time

**Fig 2:**
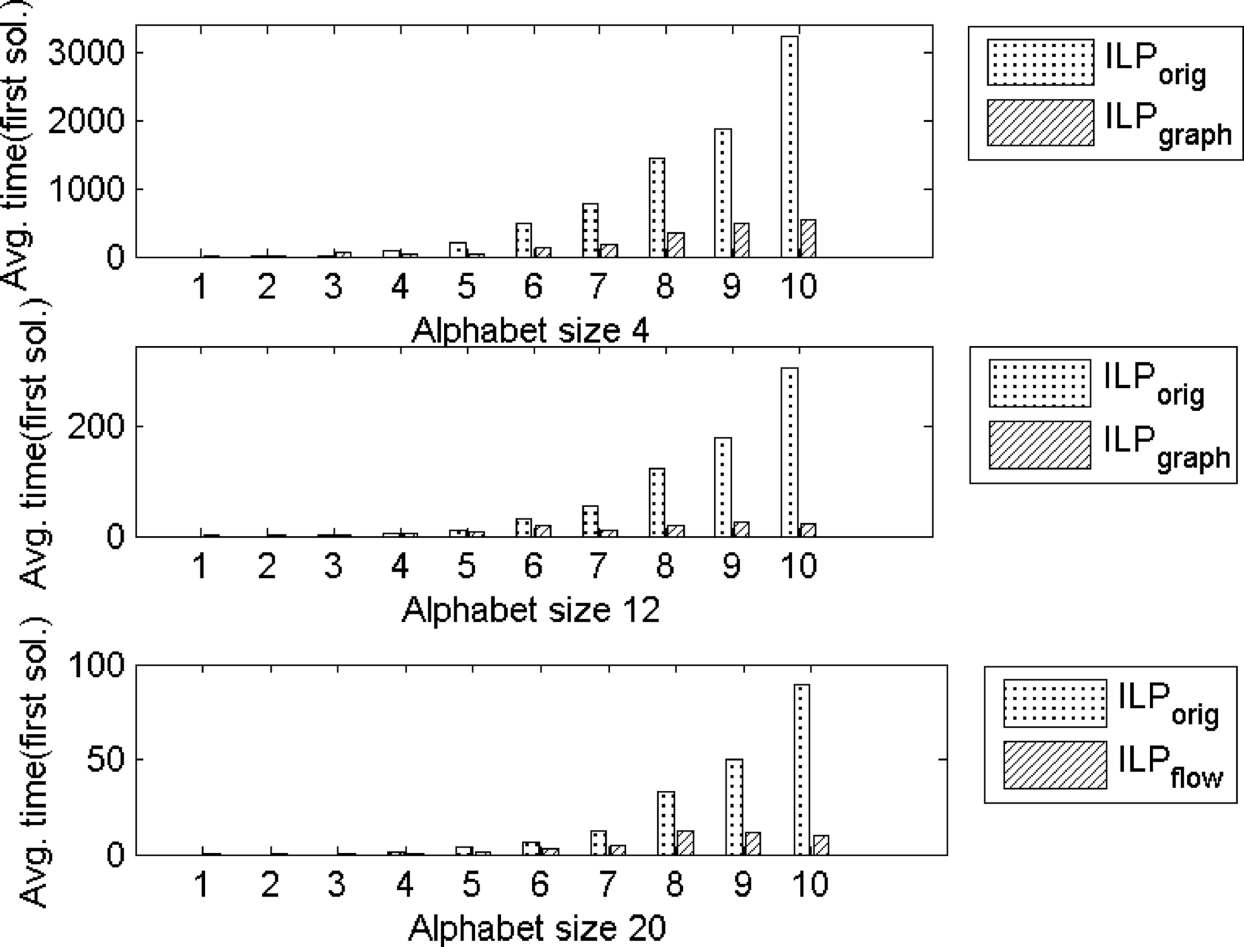
Average time for the first valid solution found by ILP_graph_ on Group4 data. Top: Alphabet size 4. Middle: Alphabet size 12. Bottom: Alphabet size 20.

**Fig 3:**
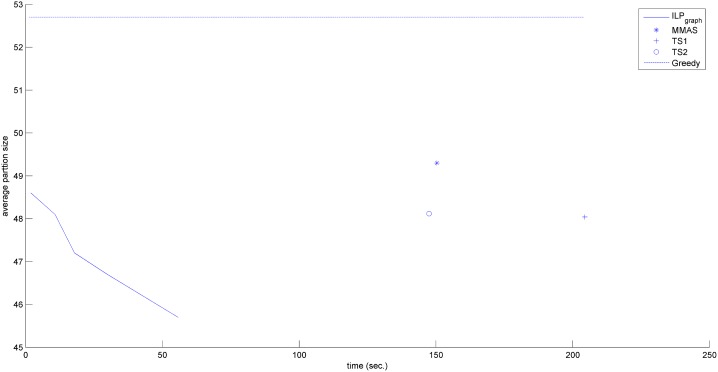
Avg. solution Vs. time comparison (Group1).

**Fig 4:**
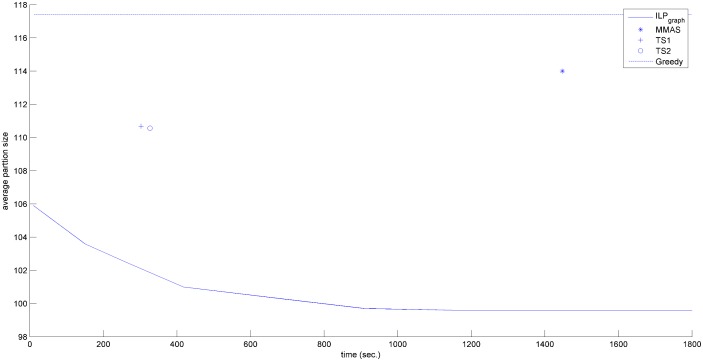
Avg. solution Vs. time comparison (Group2).

**Fig 5:**
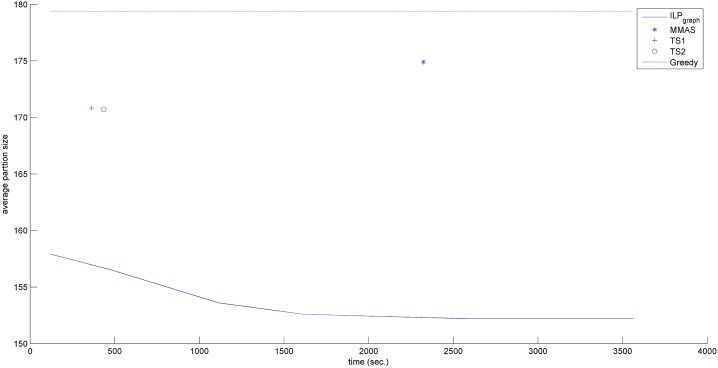
Avg. solution Vs. time comparison (Group3).

**Fig 6:**
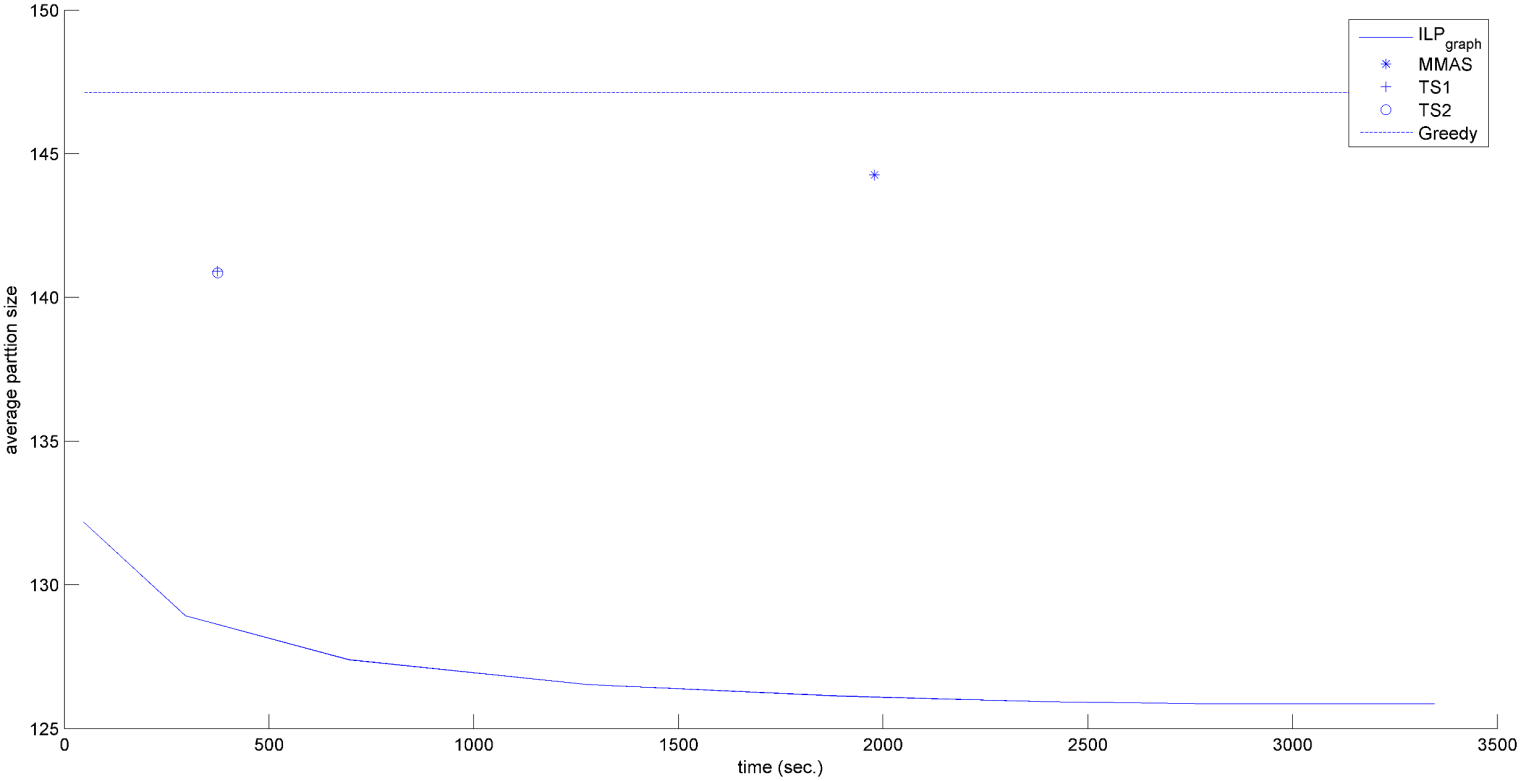
Avg. solution Vs. time comparison (Real).

In the previous section, we have shown that ILP_graph_ provides much better partition size. In this section we will explore the running time of ILP_graph_. It is clear from Tables 1–3 that ILP_graph_ achieves faster solution in most of the cases even running on a slower processor having lesser memory. This is also true for the first valid solution it provides. Fig 2 shows the comparison of the average first valid solution time for three groups based on the alphabet size in Group4 dataset. From this figure it is clear that ILP_graph_ finds the first valid solution faster than ILP_orig_ and the difference in the running time becomes more apparent as the length of the string increases. Now we concentrate on comparing the running time of ILP_graph_ with the other four approaches. The running times of the two tree search algorithms (referred to as TS1 and TS2) are taken from [15]. The running time of MMAS is taken from [19]. The Greedy algorithm is very fast. It gives the output within few seconds. So, in the analysis, we will assume that the output of Greedy algorithm is readily available even at the beginning of the simulation. We have recorded the primal solution (partition size) of our algorithm periodically. Figs 3–6 show the detailed runtime comparison among the algorithms for Group1, Group2, Group3 and Real datasets respectively. For each group we have shown the average partition size dynamics with respect to time. The three points (“*”,“+”,“o”) in each of the figures are the plots of average partition size vs. the average time needed to achieve that partition size for MMAS, TS1 and TS2 approach respectively (data taken from [13], [19]). The dashed line represents the Greedy partition size.

Although the reported time of ILP_graph_ (in Table 1) is higher than that of Greedy, TS1 and TS2 approaches in some instances but from the Figs 3–6, it can be easily observed that the ILP_graph_ algorithm reaches to better solutions much earlier. From the figures it is clear that ILP_graph_ is better than Greedy at any stage of time. Even if we stop the algorithm at or earlier than the average runtime of MMAS, TS1 or TS2, the ILP_graph_ provides better solutions.

## 6 Discussion

**Fig 7:**
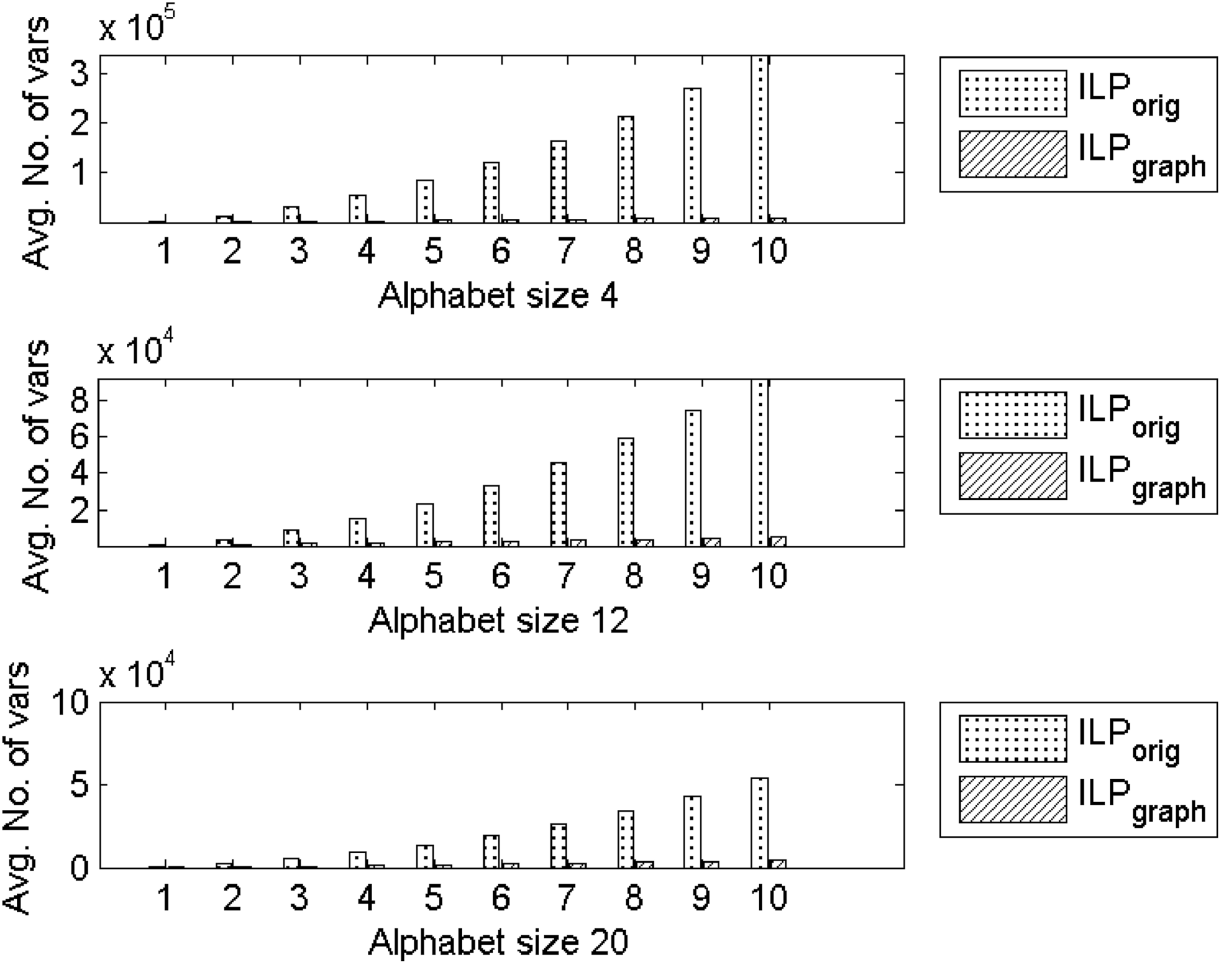
Comparison of average number of variables between ILP_orig_ and ILP_graph_ for Group4 dataset. Top: Alphabet size 4. Middle: Alphabet size 12. Bottom: Alphabet size 20.

At this point a brief discussion on the number of variables in the two ILP formulations, namely, ILP_orig_ and ILP_graph_, is in order. In Fig 7, we show the comparison of the number of variables between the two formulations for Group4 dataset. Although both formulations have *O*(*n* ^2^) variables, we observe a significant decrease in the number of variables in ILP_graph_ than ILP_orig_. The average improvement in the number of variables are reported in the last column of Tables 2 and 3 for Group4 and Group5 datasets respectively. For the Group4 dataset the maximum and minimum percentage of decrease in the number of variables are 97.06% and 57.55% with the average improvement of 88.24% while for the Group5 dataset the maximum and minimum are 98.22% and 96.48% with an average of 97.52%.

The drastic improvement in the number of variables for ILP_graph_ and the lack thereof for ILP_orig_ can easily be understood by analyzing the variable set of the two formulations. ILP_orig_ is based on *common blocks*. A *common block* *b* of two strings (*S*, *T*) is defined in [14] as a triple (*t*, *k*_1_, *k*_2_). Here *t* is a common substring of (*S*, *T*) that appeared at position *k*_1_ of *S* and *k*_2_ of *T* where 0 ≤ *k*_1_, *k*_2_ ≤ *n* − 1. *B* = {*B*_1_, *B*_2_,…*B*_*m*_} is the (ordered) set of all common blocks of (*S*, *T*). This set is the variable set of ILP_orig_. For an example, if (*S*, *T*) = (**aaagggccc**,**gggaaaccc**), then the number of common blocks would be 42. To find this, first concentrate on a common substring from *S* and *T*, namely **aaa**. The common blocks resulting from this common substring are, *B* = {[**a**, 0, 3], [**a**, 0, 4], [**a**, 0, 5], [**a**, 1, 3], [**a**, 1, 4], [**a**, 1, 5], [**a**, 2, 3], [**a**, 2, 4], [**a**, 2, 5], [**aa**, 0, 3], [**aa**, 0, 4], [**aa**, 1, 3], [**aa**, 1, 4], [**aaa**, 0, 3]}. Similar common blocks can be computed for the other two common substrings (**ggg** and **ccc**) too. On the other hand the number of variables in ILP_graph_ depends on the number of edges in the common substring graph. Thus, for the above example, if we construct the common substring graph on *S*, we have 18 edge blocks, *E* = {[*S*, 0, 0], [*S*, 0, 1], [*S*, 0, 2], [*S*, 1, 1], [*S*, 1, 2], [*S*, 2, 2], [*S*, 3, 3], [*S*, 3, 4], [*S*, 3, 5], [*S*, 4, 4], [*S*, 4, 5], [*S*, 5, 5], [*S*, 6, 6], [*S*, 6, 7], [*S*, 6, 8], [*S*, 7, 7], [*S*, 7, 8], [*S*, 8, 8]}. Thus ILP_graph_ reduces the number of variables significantly.

## 7 Conclusion and Future works

In this paper, we have presented an ILP formulation for the MCSP problem. We have conducted extensive experiments and compared the results with the state-of-the-art algorithms in the literature. The results clearly indicate that the ILP formulation is effective and provides excellent results. The observations of Section 5.4 bear important research directions. The research on this field should now be focussed on finding MCSP for larger instances in reasonable time. As ILP_graph_ provides better solution faster than the other competitive approaches, one idea is to stop the solver as soon as it gets the first solution. This solution or possibly a set thereof can be used as the initial solution(s) for existing and new meta-heuristic approaches developed to solve this problem including the ones reported in [4] and [13]. Another research direction could be as follows. So, far MCSP has been studied mostly in the context of operations research. However, it has important applications in genome comparison and rearrangement. So, datases from comparative genomics applications could be gathered for further experimental analysis and comparison with relevant algorithms (e.g., [10]) in the field of computational biology.

## Supporting Information

S1 Dataset Group1 dataset. The text file contains 10 instances in pair for Group1 dataset.
(TXT)

S2 Dataset Group2 dataset. The text file contains 10 instances in pair for Group2 dataset.
(TXT)

S3 Dataset Group3 dataset. The text file contains 10 instances in pair for Group3 dataset.
(TXT)

S4 Dataset Group4 dataset. The compressed folder contains 300 files each containing an instance of lengths from 100 to 1000 separating by alphabet size.
(TGZ)

S5 Dataset Group5 dataset. The compressed folder contains 7 files each consisting an instance of lengths from 800 to 2000.
(TGZ)

S6 Dataset Real dataset. The text file contains 10 instances in pair for Real dataset.
(TXT)

S7 Dataset 3000 length instance. The text file contains an instance of length 3000.
(TXT)
